# *Igf2* regulates early postnatal DPP4^+^ preadipocyte pool expansion

**DOI:** 10.1101/gad.352710.125

**Published:** 2025-12-01

**Authors:** Irem Altun, Khanh Ho Diep Vo, Safal Walia, Xiaocheng Yan, Inderjeet Singh, Ruth Karlina, Viktorian Miok, Lingru Kang, Valentin Kenneth Reichenbach, Andreas Israel, Dominik Lutter, Fabiana Perrochi, Siegfried Ussar

**Affiliations:** 1 RU Adipocytes and Metabolism, Helmholtz Diabetes Center, Helmholtz Zentrum München, German Research Center for Environmental Health GmbH, 85764 Neuherberg, Germany;; 2 German Center for Diabetes Research (DZD), 85764 Neuherberg, Germany;; 3 Institute for Diabetes and Obesity, Helmholtz Diabetes Center, Helmholtz Zentrum München, German Research Center for Environmental Health GmbH, 85764 Neuherberg, Germany;; 4 School of Medicine and Health, Technical University of Munich, 81675 Munich, Germany;; 5 Munich Cluster for Systems Neurology, 81377 Munich, Germany;; 6 Institute of Neuronal Cell Biology, Technical University of Munich, 80802 Munich, Germany

**Keywords:** DPP4^+^, hyperplasia, IGF2, progenitor cells, adipose tissue development, preadipocyte

## Abstract

Adipose tissue is rapidly expanding early in life. Elucidating the queues facilitating this process will advance our understanding of metabolically healthy obesity. Using single-cell RNA sequencing, we identified compositional differences of prewean and adult murine subcutaneous adipose tissue. We identified a dipeptidyl peptidase-4 (*Dpp4*)-positive precursor population residing in the reticular interstitium of subcutaneous adipose expressing insulin growth factor 2 (*Igf2*) in prewean mice. We show that IGF2 drives proliferation rather than differentiation in these cells. Moreover, loss of *Igf2* in *Dpp4*^+^ progenitor cells promotes adipogenesis. Our findings unravel the temporally restricted expression of *Igf2* to promote preadipocyte expansion.

## Abstract (precis)

In this study, Altun et al. compare the composition of adipose tissue from postnatal and adult mice, identifying a key role for insulin growth factor 2 (IGF2) in the proliferation of adipocyte progenitors. They show that the loss of preadipocyte IGF2 favors adipogenesis, revealing that the temporal dynamics of IGF2 may be critical in balancing proliferation and differentiation programs during adipose expansion and maturation during early life.

White adipose tissue (WAT) development begins in utero and continues after birth (Wang et al. 2013). In humans, a rapid and massive expansion of the adipose tissue, mainly by the increase in adipocyte size, peaks within the first year of life, followed by a significant decrease after 2 years postpartum (Knittle et al. 1979). In line with this, murine subcutaneous adipose tissue mass, relative to body weight, peaks at 12 days postpartum and decreases proportionally during weaning (Tsukada et al. 2023). Differences in adipocyte size before, during, and after weaning further support developmental changes and remodeling of the tissue during early life (Zhang et al. 2022; Tsukada et al. 2023; Qian et al. 2024). These reconfigurations are critical, as the continued expansion of adipose tissue into adolescence results in obesity in adulthood (Knittle et al. 1979). However, the regulation of this initial metabolically healthy adipose tissue expansion early in life remains incompletely understood (Altun et al. 2022).

WAT is a heterogenous endocrine organ. It consists of immune, adipose progenitor (APC), endothelial, and neuronal cell populations (Suwandhi et al. 2021; Altun et al. 2022; Emont et al. 2022). Several studies identified distinct APC subtypes with different functions in adipose tissue physiology. Sakers et al. (2022) defined APCs as highly proliferative cells with stem-like properties that maintain the progenitor niche in the tissue (Altun et al. 2022). Merrick et al. (2019) identified a DPP4^+^ progenitor cell population that gives rise to committed preadipocytes. These cells, residing in the reticular interstitium (RI), an area surrounding adipose tissue, have an increased proliferation rate. In addition, some precursors directly promote adipogenesis (Rodeheffer et al. 2008; Merrick et al. 2019; Altun et al. 2022). However, what regulates the proliferative capacity of these cells and induces commitment to differentiation needs further investigation.

Insulin growth factor 2 (IGF2) is a secreted protein that is essential for fetal growth (DeChiara et al. 1991; Sandovici et al. 2022). IGF2 is a member of the insulin superfamily and can activate both insulin receptor (InsR) and IGF1 receptor (IGF1R) signaling pathways (Frasca et al. 1999; Blyth et al. 2022). Both pathways have been described as regulators of adipose tissue development and metabolism (Blüher et al. 2002; Boucher et al. 2012). Recently, Rondini et al. (2021) identified, among other genes, Igf2 as highly expressed in subcutaneous adipose tissue of young (up to postnatal day 18) but not adult mice. Even though IGF2 supplementation was shown to regulate adipogenesis in a depot-dependent manner, the target APC population or the direct function of *Igf2* is not well understood (Alfares et al. 2018).

Here, we compared compositional differences of subcutaneous white adipose tissue (scWAT) between the prewean and adult state in mice to discover cell populations or factors regulating healthy adipose tissue expansion. We identified a subpopulation of progenitor cells that are *Dpp4*^+^*Igf2*^+^ residing at the RI in prewean mice. DPP4^+^ preadipocytes lose *Igf2* expression later in life, together with their high proliferative capacity. Furthermore, loss of *Igf2* function induces differentiation of the DPP4^+^ preadipocytes. Thus, our results propose a mechanism for the regulation and maintenance of progenitor cell expansion.

## Results and Discussion

### scRNA sequencing analysis reveals *Igf2* as the most differentially expressed gene between prewean and adult scWAT preadipocytes

**Figure 1.**
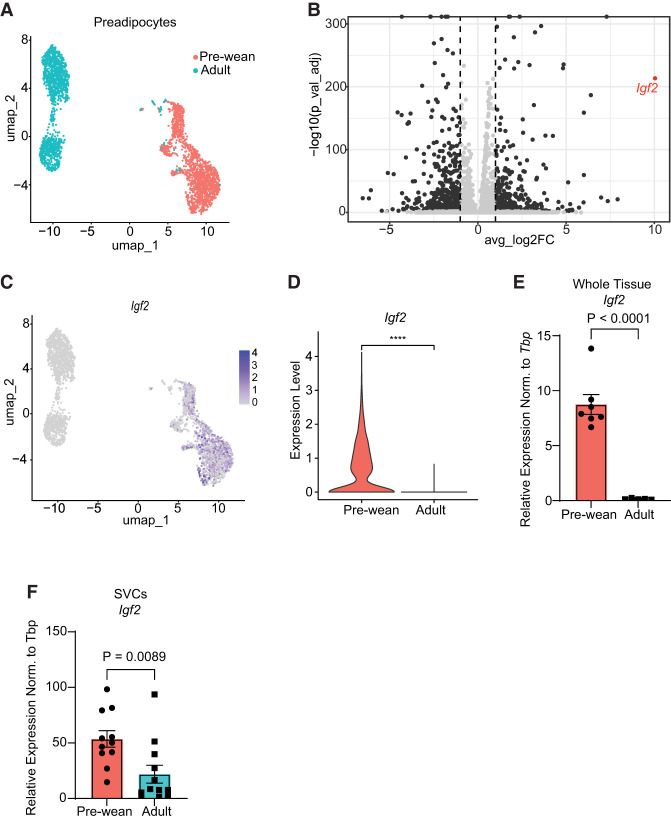
scRNA-seq analysis reveals *Igf2* as the most abundantly expressed gene in prewean scWAT preadipocytes. (*A*) Projection of two age groups (prewean and adult) on scWAT preadipocytes, shown as a UMAP plot. (*B*) Differential gene expression comparing prewean versus adult scWAT preadipocytes. The red dot represents *Igf2*. (*C*) UMAP plot showing the projection of *Igf2* on scWAT preadipocytes. (*D*) Violin plot showing *Igf2* expression in two age groups. (*E*,*F*) RT-qPCR analysis of *Igf2* expression in whole tissue (*n* = 7 prewean and *n* = 5 adult) (*E*) and isolated SVCs (*n* = 11 prewean and *n* = 12 adult) (*F*). Data are shown as mean ± SEM.

We previously reported single-cell RNA sequencing (scRNA-seq) experiments comparing prewean and adult scWAT stromal vascular cells (SVCs) (Suwandhi et al. 2021). This analysis revealed a distinct separation of *Pdgfra*^*+*^ adipose stromal and progenitor cells (ASPCs) between the two age groups (Supplemental Fig. S1A,B; Suwandhi et al. 2021). We further analyzed these data to identify new subsets of ASPCs or individual genes contributing to adipose tissue expansion via hyperplasia. Our analysis revealed 18 cell clusters consisting of a variety of cell types, including immune cells and endothelial cells (Supplemental Fig. S1B). Clusters 1, 2, 5, and 8 expressed elevated levels of ASPC markers (*Pdgfra*, *Cd34*, and *Dlk1*) (Supplemental Fig. S1C), which were selected for further analysis (Fig. 1A). Differential gene expression analysis of prewean and adult scWAT ASPCs revealed *Igf2* as the most differentially expressed gene in prewean compared with adult ASPCs (Fig 1B,C; Supplemental Fig S1D). Some additional minor expression of Igf2 was observed in endothelial cells (Supplemental Fig S1E). Further analysis confirmed that *Igf2* mRNA levels were significantly lower in scWAT ASPCs of adult mice compared with prewean mice (Fig. 1D,F). The much higher expression of *Igf2* in SVCs compared with the whole tissue further suggests only a minor contribution of *Igf2* expression in mature adipocytes. We then assessed the IGF2 signaling pathway in ASPCs by stimulating cultured primary ASPCs with 10 nM IGF2 in vitro. This resulted in the upregulation of AKT and ERK phosphorylation after 10 min compared with control conditions (Supplemental Fig. S1F). The activation of these pathways was mediated by activation of both the IGF1R and the InsR, as shown by phosphorylated tyrosines detected after stimulation with IGF2 in both receptors (Supplemental Fig. S1G,H).

### IGF2 does not influence the differentiation of primary ASPCs in vitro

**Figure 2.**
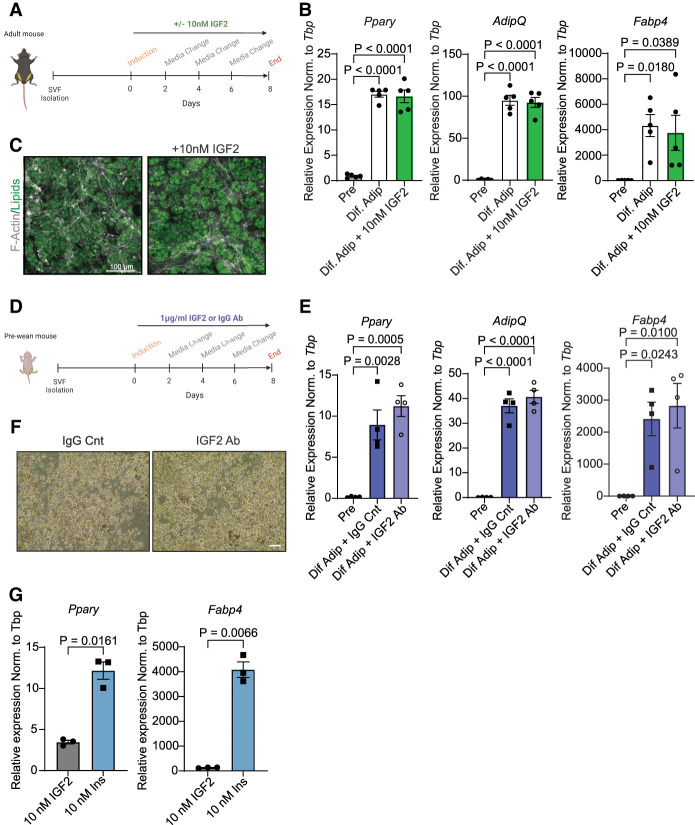
GF2 supplementation does not affect differentiation of scWAT preadipocytes in vitro. (*A*) Schematic illustration of 10 nM IGF2 supplementation of cultured primary adult scWAT preadipocytes during the whole course of differentiation. (*B*) mRNA levels of adipogenic markers (*n* = 5). (*C*) Immunocytochemistry of lipids (green) and F-actin (gray) (*n* = 3). (*D*) Schematic illustration of 1 µg/mL IgG control or IGF2-neutralizing antibody (Ab) treatment of cultured primary prewean scWAT preadipocytes during the whole course of differentiation. (*E*) mRNA levels of adipogenic markers (*n* = 4). (*F*) Bright-field images of differentiated adipocytes treated with either IgG control or IGF2-neutralizing antibody. Scale bar, 150 µm (*n* = 4). (*G*) Insulin (100 nM) was substituted with either 10 nM IGF2 or 10 nM insulin during differentiation. mRNA levels of adipogenic markers of differentiated primary adult preadipocytes with either IGF2 or insulin (*n* = 3). (Pre) Preadipocytes, (Dif. Adip) differentiated adipocytes. Data are shown as mean ± SEM.

Both IGF1R signaling and InsR signaling play important roles in adipogenesis. Thus, we investigated the role of *Igf2* in ASPC differentiation. As shown in Figure 1F, adult ASPCs express significantly lower levels of *Igf2* compared with prewean mice. Therefore, to mimic the preweaning conditions, primary adult ASPCs were supplemented with 10 nM IGF2 throughout differentiation (days 08 [d0–d8]) (Fig. 2A). *Igf2* mRNA levels remained the same in differentiated adipocytes compared with ASPCs (Supplemental Fig. S2A). Furthermore, supplementation of IGF2 did not alter the mRNA expression levels of *Igf2* and adipogenic markers and protein levels of PPARg (Fig. 2B; Supplemental Fig. S2A,B). Additionally, lipid accumulation assessed by immunocytochemistry and Oil-Red-O (ORO) staining did not show differences in lipid accumulation (Fig. 2C; Supplemental Fig. S2C). We next tested the effects of inhibition of IGF2 on differentiation by treating primary prewean ASPCs with 1 µg/mL IGF2-neutralizing antibody during the whole course of differentiation (Fig. 2D). The specificity of the antibody was tested by treating the primary preadipocytes stimulated with 10 nM IGF2 with either neutralizing antibody or IgG control for 24 h in serum-free media. The phosphorylation of AKT was reduced with the neutralizing antibody compared with the control, implying that IGF2 signaling was at least partially blocked (Supplemental Fig. S2D). Interestingly, we observed a slight increase in the expression of adipogenic markers (*Pparg*, *AdipQ*, and *Fabp4*) in cells differentiated with IGF2-neutralizing antibody compared with IgG controls. However, this did not reach statistical significance (Fig. 2E). The same trend was observed by morphological assessment using bright-field imaging (Fig. 2F). In contrast to the adult ASPCs, Igf2 expression was significantly reduced in differentiated adipocytes of prewean mice compared with ASPCs (Supplemental Fig. S2E). However, these experiments were conducted in the presence of 100 nM insulin, which activates both the InsR and IGF1R at this concentration and could mask the effects of IGF2 supplementation. Therefore, we tested the role of IGF2 in adipogenesis by differentiating primary ASPCs from adult scWAT by substituting 100 nM insulin in differentiation medium with either 10 nM IGF2 or 10 nM insulin. Cells treated with IGF2 differentiated significantly less compared with insulin-treated cells, as shown by *Pparg* and *Fabp4* expression (Fig. 2G). There was no significant difference in *Igf2* and *AdipQ* mRNA levels between conditions (Supplemental Fig. S2E). These findings indicated that insulin is essential for preadipocyte differentiation and that IGF2 cannot enhance differentiation.

### IGF2 enhances proliferation of Dpp4^+^ preadipocytes

IGF2 is a fetal growth hormone and highly abundant in fetal serum. To mitigate the potentially high endogenous IGF2 levels in FBS that may interfere with the effects of exogenously supplemented IGF2, we investigated alternative serum sources. Previous studies have shown that circulating IGF2 levels vary with age in mice. Consequently, our findings revealed that although serum insulin levels remained constant, IGF2 levels were significantly higher in prewean mice (239.4 ng/mL ± 28.2 ng/mL) compared with adult mice (9.0 ng/mL ± 0.6 ng/mL) (Supplemental Fig. S3A,B). Additionally, prewean ASPCs cultured overnight in serum-free media secreted slightly more IGF2 than adult ASPCs, though this difference was not statistically significant (Supplemental Fig. S3C). Based on these results, we proceeded to differentiate primary ASPCs in media containing 1% adult mouse serum (MS), following the protocol outlined in Figure 1A. Interestingly, *Igf2* expression levels were significantly downregulated in adult ASPCs differentiated in mouse serum (Supplemental Fig. S3D). However, the differentiation capacity, as measured by mRNA levels of adipogenic markers, lipid content, immunocytochemistry of lipids, and protein levels of PPARγ, was unchanged in cells supplemented with 10 nM IGF2 compared with controls (Supplemental Fig. S3E–H). A similar outcome was observed when prewean ASPCs were treated with IGF2-neutralizing antibody and differentiated in mouse serum compared with IgG control-treated cells based on following the protocol outlined in Figure 1D (Supplemental Fig. S3I,J). Thus, despite the varying culture conditions, IGF2 does not directly influence adipogenesis.

**Figure 3.**
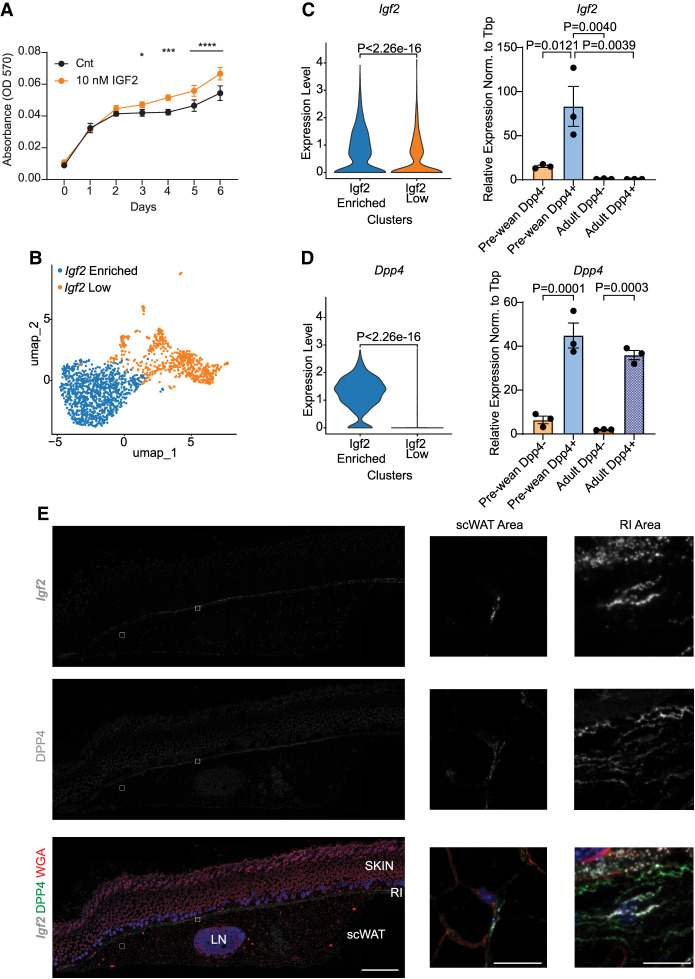
DPP4^+^ preadipocytes express significantly high levels of *Igf2*. (*A*) MTT assay of primary adult scWAT preadipocytes cultured with 1% adult mice mouse serum and supplemented with or without 10 nM IGF2 for 7 days (*n* = 5). (*B*) Reclustering of prewean preadipocytes only, shown as a UMAP plot. (*C*) Violin plot of *Igf2* expression between two clusters identified in *B* (*left*) and bar graph of mRNA expression levels in DPP4 sorted cells from isolated prewean and adult SVCs (*right*) (*n* = 3). (*D*) Violin plot of *Dpp4* expression between two clusters identified in *B* (*left*) and bar graph of mRNA expression levels in DPP4 sorted cells from isolated prewean and adult SVCs (*right*) (*n* = 3). (*E*) Fluorescent in situ hybridization by RNAscope of *Igf2* (gray) and immunohistochemistry of DPP4 (green), WGA (red), and DAPI (blue) were performed on prewean scWAT isolated with skin (*n* = 4). Scale bars: *left*, 500 µm; *right*, 25 µm. The tile scan images were taken with a 40× objective. (*) *P* = 0.0222, (***) *P* = 0.0002, (****) *P* < 0.0001. Data are shown as mean ± SEM.

Because IGF2 is a growth factor, we next tested whether it induces proliferation rather than differentiation. Primary adult preadipocytes were supplemented with 10 nM IGF2 for 7 days. MTT assays revealed significantly higher proliferation capacity of the cells supplemented with 10 nM IGF2 compared with controls using 1% adult mouse serum (Fig. 3A).

To obtain more detailed insights into the prewean *Pdgfra*^*+*^ ASPC populations expressing *Igf2*, we reclustered these cells by *Igf2*-enriched and low-expressing cells (Fig. 3B,C). Differential gene expression analysis comparing *Igf2*-enriched versus low cell clusters revealed 387 upregulated and 466 downregulated genes. Gene set enrichment analysis for gene ontology (GO) terms revealed that *Igf2*-enriched cells express high levels of genes associated with “positive regulation of cell population proliferation” and “extracellular matrix organization,” suggesting a potential role of *Igf2* in ASPC niche formation or maintenance (Supplemental Fig. S4A). Furthermore, scRNA-seq analysis revealed *Dpp4* as one of the most highly expressed genes in the *Igf2*-enriched cell population. This finding was further validated by MACS sorting of DPP4^+^ preadipocytes, which showed significantly higher levels of *Igf2* expression compared with DPP4^−^ cells in prewean mice (Fig. 3C,D). Analysis of Igf1R and InsR expression in these cells did not show differences in InsR expression but a trend for increased Igf1R expression in prewean mice irrespective of Dpp4 expression (Supplemental Fig. S4B). In line with our above results, the *Igf2* expression was absent in adult DPP4^+^ cells (Fig. 3D). Moreover, supplementation of differentiation medium with 10 nM IGF2 throughout differentiation (d0–d8) did not increase differentiation as assayed by analyzing the mRNA expression levels of adipogenic markers in either adult DPP4^+^ or DPP4^−^ cells (Supplemental Fig. S4C). Proliferation assays with adult DPP4^+^ and DPP4^−^ cells in the absence or presence of IGF2 revealed a moderate increase in proliferation of DPP4^−^ cells but no effects on DPP4^+^ cells (Supplemental Fig. S4D), indicating that IGF2 plays a less important role in regulating adult DPP4^+^ cell proliferation.

FISH and immunohistochemistry analysis showed that DPP4^+^*Igf2*^*+*^ cells reside at the reticular interstitium (RI) region of the prewean scWAT (Fig. 3E), which is critical for tissue expansion and regeneration (Merrick et al. 2019). However, we observed additional DPP4^+^ cells close to the lymph node, which morphologically appeared more like beige, multilocular adipocytes.

### Loss of *Igf2* promotes differentiation of DPP4^+^ SVCs

**Figure 4.**
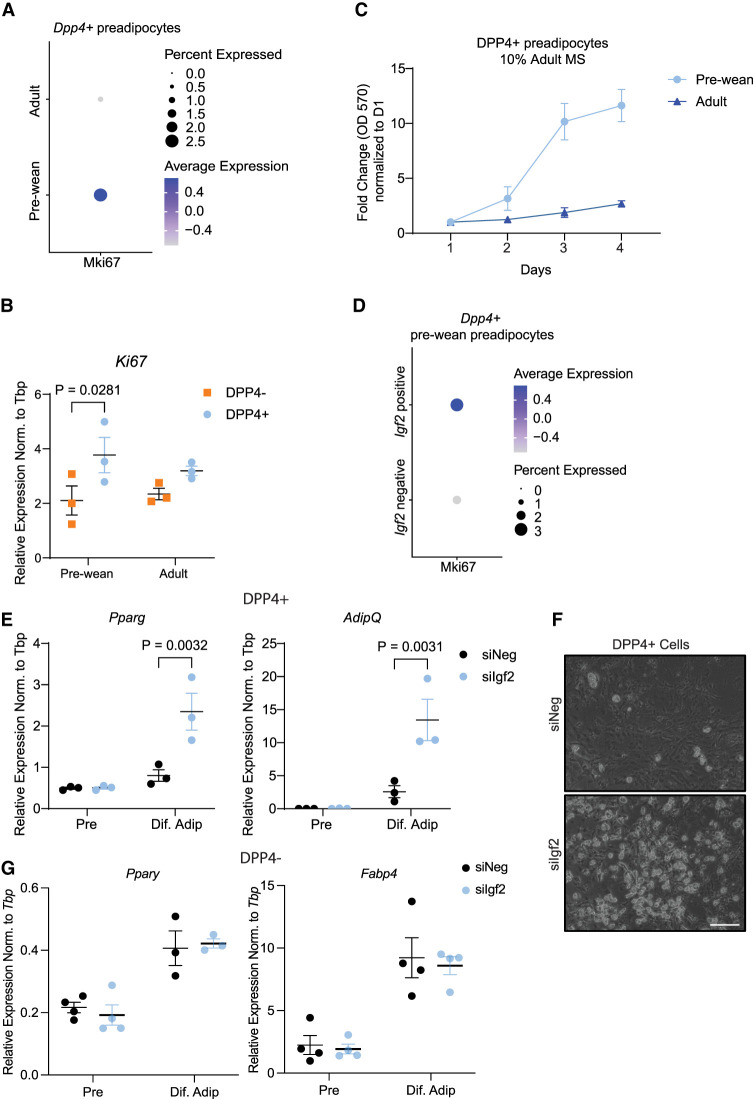
Loss of *Igf2* in DPP4^+^ cells induces preadipocyte differentiation. (*A*) Dot plot of *Ki67* expression in prewean and adult *Dpp4*^+^ preadipocytes. (*B*) mRNA expression of *Ki67* in MACS-sorted DPP4^+^ and DPP4^−^ prewean and adult cells (*n* = 3). (*C*) MTT assay of DPP4^+^ primary preadipocytes isolated from prewean and adult mouse scWAT (*n* = 2). (*D*) Dot plot of Ki67 in *Dpp4*^+^ prewean preadipocytes comparing *Igf2*-positive and *Igf2*-negative cells. *Igf2* was knocked down (siIgf2) in prewean DPP4^+^ primary preadipocytes. (*E*) mRNA expression of adipogenic markers (*n* = 3). (*F*) Bright-field images of *Igf2* knockdown and control differentiated prewean preadipocytes (*n* = 4). Scale bar, 150 μm. (*G*) mRNA expression of adipogenic markers in DPP4^−^ cells (*n* = 3). Data are shown as mean ± SEM.

Given our data indicating that IGF2 increases cell proliferation and prior research characterizing DPP4^+^ preadipocytes as highly proliferative (Merrick et al. 2019), we explored whether *Igf2* helps maintain proliferation of DPP4^+^ cells early in life. Analysis of the scRNA-seq data from prewean DPP4^+^ cells revealed higher expression of the proliferation marker *Ki67* compared with adult DPP4^+^ cells (Fig. 4A). qPCR analysis did not show a difference in *Ki67* expression between prewean and adult DPP4^+^ cells, whereas we observed a significant difference between prewean DPP4^+^ and DPP4^−^ cells (Fig. 4B). Furthermore, we found that prewean DPP4^+^ cells proliferated at a higher rate than adult cells (Fig. 4C). Moreover, *Igf2*^+^ cells express higher *Ki67* levels compared with *Igf2*^−^ cells (Fig. 4D). These findings suggest that *Igf2* expression promotes DPP4 cell proliferation. However, blocking endogenous IGF2 with an IGF2-blocking antibody did not reduce proliferation (Supplemental Fig. S4E) Thus, we further tested whether *Igf2* is important for the proliferation of DPP4^+^ cells by knocking down Igf2 (siIgf2) in primary DPP4^+^ and DPP4^−^ cells from prewean scWAT using small interferening RNA (siRNA) (Supplemental Fig. S4F). Evaluation of adipogenic markers by RT-qPCR and bright-field images revealed that siIgf2 DPP4^+^ preadipocytes differentiated significantly more compared with control cells (siNeg) (Fig. 4E,F), whereas this was not observed for DPP4^−^ cells (Fig. 4G). These findings strongly support our finding that *Igf2* is essential for DPP4^+^ cells to maintain their proliferative capacity and precursor nature early in life. However, conditional deletion of Igf2 in DPP4^+^ cells would be required to fully address the impact of Igf2 on proliferation.

Adipose tissue development and expansion occur via hyperplasia and hypertrophy (Wang et al. 2013). Subcutaneous adipocyte hyperplasia is associated with healthier metabolism (Vishvanath and Gupta 2019). This process is tightly regulated by preadipocyte proliferation and de novo adipogenesis. Because preadipocyte populations tightly regulate this process, it is critical to understand different subtypes present in the adipose tissue and the microenvironment that they create to regulate tissue expansion (Altun et al. 2022).

To this end, we previously described scRNA-seq analysis of scWAT SVCs comparing prewean and adult mice, aiming to identify compositional differences during developmental states that positively regulate adipose tissue homeostasis. In this study, we reanalyzed this data set focusing only on *Pdgfra*^*+*^ preadipocytes, as the most distinct separation by age was observed in this cell population (Suwandhi et al. 2021). As a result, we identified *Igf2* as the most abundantly expressed gene in prewean scWAT ASPCs, with some expression in prewean endothelial cells. This was further confirmed at mRNA levels in whole tissue and isolated SVCs. Its significant decrease in adult mice, as also reported previously (Rondini et al. 2021), suggests a critical developmental function of the gene in adipose tissue development. Additionally, even though IGF2 is an important fetal growth promoter, the postnatal role of the protein is not well understood. Consistent with previous data, we found that 10 nM IGF2 activates PI3K/AKT and MAPK/ERK pathways through IGF1R and InsR in primary preadipocytes (Frasca et al. 1999; Blyth et al. 2022). These two receptors have different physiological roles in the cells; while IGF1R regulates mitogenic activity and is expressed higher in preadipocytes, InsR is essential for metabolic activity and is expressed more in adipocytes (Boucher et al. 2010). Furthermore, loss of both receptors in preadipocytes severely affects differentiation (Boucher et al. 2010). It will be interesting to study the exact downstream effects of IGF2-mediated signaling in prewean DPP4^+^ and the potential differences between prewean and adult DPP4^+^ cells with regard to IGF2 signaling. Currently, these studies are limited by the low cell numbers of primary cells from tissue and the absence of suitable immortalized cell models.

First, the role of IGF2 in adipocyte differentiation was investigated by supplementing adult ASPCs with IGF2 to mimic the high *Igf2* expression in prewean mice, whereas prewean preadipocytes were treated with neutralizing antibody to block receptor–ligand interaction. Even though blockage of IGF2 and receptor interaction showed a slight increase in mRNA levels of adipogenic markers, there were no significant differences between control and treatment conditions in both approaches. Moreover, this antibody did not reduce proliferation of prewean DPP4^+^ cells, indicating that complete blockage would be necessary, which could be limited by matrix-bound IGF2.

A study of normal-weight children showed that IGF2 supplementation enhances scWAT preadipocyte differentiation while inhibiting visceral preadipocyte differentiation in a dose-dependent manner (7.5 and 62.5 ng/mL) (Alfares et al. 2018). Additionally, rat adipose stem cells showed that supplementation of 100 ng/mL IGF2 only under 1% FBS promotes adipocytes’ differentiation and self-renewal capacity through IGF1R and InsR but has no effect using 10% FBS (Wang et al. 2019). Differences between previous studies and our results could be due to species differences or the fact that white adipose tissue SVCs are heterogenous and that cellular composition of isolated preadipocytes can vary between studies based on age and isolation site. This has been discussed previously (Altun et al. 2022; Palani et al. 2023). Moreover, because IGF2 is a fetal growth factor, exogenous protein levels present in fetal serum could impact study results (Tu et al. 2018). To overcome these limitations and uncertainties, we substituted FBS with adult mouse serum that contained low levels of IGF2. We did not observe differences in the differentiation of the ASPCs between treatment and control groups in both prewean and adult cells. Along with these findings, substitution of insulin in the differentiation media with IGF2 significantly reduced mature adipocyte gene expression and differentiation of preadipocytes. Thus, our data strongly support the conclusion that IGF2 does not enhance adipogenesis of primary murine preadipocytes.

Several studies have highlighted the role of IGF2 in promoting proliferation in various cell types (Wang et al. 2019; Sandovici et al. 2022). In line with this, our results demonstrated that supplementation of adult preadipocytes that express low levels of *Igf2* with 10 nM IGF2 enhanced their proliferation. scRNA-seq analysis revealed that *Igf2*^*+*^ cells express significantly high levels of *Dpp4* early in life. Consistent with the results, these cells lost *Igf2* expression in adulthood. Merrick et al. (2019) identified the DPP4^+^ cell population as highly proliferative and multipotent precursors; however, the mechanism behind this is not well understood. Proliferation assays showed that isolated adult DPP4^+^ cells proliferate significantly less compared with prewean cells. We showed that one of the significant differences between these two cell populations is the *Igf2* expression. However, we cannot exclude additional physiological changes during development. Thus, to better evaluate the function of *Igf2* specifically in DPP4^+^ cells, we performed a loss-of-function study with small interferening RNA (siRNA) in sorted prewean DPP4^+^ and DPP4^−^ cells. *Igf2* knockdown DPP4^+^ cells expressed significantly higher levels of adipogenic markers and showed more lipid accumulation compared with control knockdown cells, whereas this was not seen in DPP4^−^ cells. The same phenomenon has been reported with *Dpp4* knockdown, which induced human preadipocyte differentiation (Hatzmann et al. 2022). These findings demonstrate that *Dpp4* expression and *Igf2* expression together potentially regulate proliferation and expansion of the DPP4^+^ precursor population and that loss of expression of either one of them commits cells to differentiation. However, this seems to be a function restricted to initial adipose tissue expansion, as data from Emont et al. (2022) did not show expression of Igf2 upon HFD feeding of mice.

In conclusion, we show for the first time a key function of *Igf2* expression in a previously characterized adipose precursor cell population. Our findings suggest that *Igf2* expression is essential in maintaining the proliferative capacity of DPP4^+^ cells early in life to serve as a reservoir for adipose progenitor pools and potentially contribute to tissue development and expansion at the reticular interstitum region, which surrounds the scWAT and is rich in collagen and fibers. It will be interesting to study whether the same cell population and mechanism also exists in perigonadal/visceral adipose tissue, which develops later in mice but shows great plasticity later in life. Thus, this study allows better understanding of developmental mechanisms in adipose progenitor populations found in a distinct location to allow targeting of new therapeutic approaches for promoting metabolically healthy adipose tissue expansion.

## Materials and methods

### Mice

Prewean (15 day old ± 2 days) and adult (58 day old ± 2 days) C57Bl6 wild-type mice were housed at a constant ambient temperature of 22°C ± 2°C at 45%–65% humidity with a 12 h light–dark cycle and ad libitum access to standard chow diet (Altromin 1314) and water. During the sacrifice of animals, blood was withdrawn and kept for 15 min at room temperature prior to incubation on ice. Mouse serum (MS) was obtained by centrifugation at 10,000*g* for 5 min at room temperature. Serum from 27 mice was pooled, filtered through a 0.45 µm filter, and stored at −80°C for cell culture experiments. All animal procedures were performed under the guidance and approval of German Animal Welfare Law and the district government of Upper Bavaria, Bavaria, Germany.

### Primary cell culture

Primary cells were isolated from subcutaneous adipose tissue (scWAT) of prewean and adult C57Bl6 wild-type male mice. Freshly isolated tissues were chopped into small pieces and digested in digestion solution (Dulbecco's modified Eagle's medium [DMEM] high glucose + GlutaMAX [Gibco] containing 1% BSA and 1 mg/mL collagenase type I [Life Technologies]) with shaking at 1000 rpm for 30–40 min at 37°C. Collagenase type I was substituted with collagenase type IV (Life Technologies) to isolate DPP4^+^ preadipocytes. The suspension was then filtered through a 100 µm strainer. Cells were centrifuged at 500*g* for 5 min at room temperature. Pelleted SVCs were washed once with PBS and either stored at −80°C for total RNA isolation or cultured in normal growth media (DMEM, 10% fetal bovine serum [FBS], 1% penicillin and streptomycin [PenStrep], 0.1 mg/mL normocin [Invitrogen]) at 37°C and 5% CO_2_.

#### Preadipocyte differentiation

Preadipocytes were seeded and cultured to 90%–100% confluence. Confluent preadipocytes were induced (day 0 [d0]) in DMEM with 10% FBS or Opti-MEM with 1% or 10% MS containing 0.5 mM 3-isobutyl-1-methylxanthine (IBMX; Sigma Aldrich), 5 µM dexamethasone (Sigma Aldrich D4902), 100 nM insulin (Sigma Aldrich), 1% PenStrep, and 0.1 mg/mL normocin. After 2 days, the induction medium was replaced by differentiation medium with only 100 nM insulin in the respective culture media conditions. The media was changed every 2 days until day 8 (d8) of differentiation. In the case of supplementation or neutralization experiments, cells were cultured with 10 nM IGF2 (Sigma Aldrich I8904) or 1 µg/mL IGF2-neutralizing antibody (IGF2 Ab; Thermo Scientific PA5-47946), respectively, from d0 to d8 of differentiation. Goat IgG (1 µg/mL; Invitrogen 31245) was used as a control for the blocking experiments, and no treatment was used as a control for supplementation experiment. Adult scWAT preadipocytes were differentiated by substituting 100 nM insulin with 10 nM IGF2 or 10 nM insulin from d0 to d8. IBMX and dexamethasone were still present between d0 and d2 of differentiation. At the end of the experiment, cells were either fixed or frozen for further analysis.

#### Igf2 knockdown

Cultured DPP4^+^ preadipocytes were transfected following a reverse transfection method as described by Isidor et al. (2016). Alternatively, cultured DPP4^+^ and DPP4^−^ preadipocytes were transfected using a forward transfection method. Briefly, preadipocytes were seeded to reach 70% confluency. A transfection mixture was prepared by combining Opti-MEM (Gibco), Lipofectamine RNAiMAX (Invitrogen), and 50 nM siRNA, followed by a 25 min incubation at room temperature. The transfection mixture was then added to the cells for 4 h, after which culture media was added and the cells were incubated for an additional 44 h before further experiments. To confirm the knockdown efficiency for Igf2, qPCR analysis was performed. To examine the effect of Igf2 knockdown on differentiation, cells were induced with 0.5 mM IBMX, 5 µM dexamethasone, and 100 nM insulin. Induction media was changed to differentiation medium containing 100 nM insulin. The media was changed every 2 days for 6–7 days. The *Igf2* siRNA (siIgf2) sequence was modified from that described by Gui et al. (2021) (mmIgf2 Frw: 5′-GGAGCUUGUUGACACGCUUCAGUU-3′ and Rev: 5′-CCUCGAACAACUGUGCGAAGUCAA-3′; IDT). A commercially available negative control siRNA (siNeg) was used (IDT 51-01-14-04).

### Single-cell RNA (scRNA) sequencing and analysis

scRNA sequencing data were published previously (Suwandhi et al. 2021) and further analyzed using R. The Cell Ranger single-cell software suite v2.1.1 from 10x Genomics was used to preprocess the data. This included demultiplexing raw BCL files to FASTQ files and mapping of reads to an indexed mm10 reference genome, which was built using the GRCm38 assembly with ensemble genome annotation release 94 and UMI counting. A gene expression count matrix along with feature barcode counts for each cell barcode was generated, resulting in 20,151 genes across 9319 cells. These matrices were then processed in R (v4.3.2) using the Seurat package (v5.1.0). The initial QC steps included filtering out cells that captured <500 expressed genes and potential doublets with a total number of detected genes >5000. The percentage of mitochondrial genes expressed was computed, and cells with >10% mitochondrial genes were discarded. The remaining cells were normalized and scaled using default parameters of Seurat functions.

For cluster generation, we performed PCA, and based on the elbow plot, the top 25 PCs were considered to generate a UMAP. Cell type was determined by examining the expression of a set of marker genes that were reviewed from the literature and observed along the clusters. Based on interesting cell types and batches, further subsets of clusters followed by coarser clustering were carried out. We considered preadipocyte clusters for the prewean mice to check expression of *Igf2-* and *Dpp4*-positive cells. For the volcano plot, differentially expressed genes were generated using the FindMArkers function of Seurat by assigning the batch type comparisons as idents. Violin plots, dot plots, and individual UMAP plots for the specific genes were performed by Seurat's VlnPlot, Dotplot, and FeaturePlot functions, respectively. We predicted classification of each cell in either G2M, S, or G1 phase by assigning scores using the CellCycleScoring function. Gene ontology (GO) analysis was performed in R using the package enrichR.

### MACS sorting of DPP4^+^ cells

Freshly isolated SVCs from scWAT of prewean and adult wild-type male mice were washed with PBS and blocked in 1% BSA and 1 mM EDTA in PBS for 10 min at 4°C. Four to six mice were pooled for each biological replicate. Cells were incubated with a DPP4 antibody (1:10; R&D Systems AF954) for 30 min, followed by addition of 15 µL of protein G magnetic beads (Miltenyi Biotec) for 30 min. MS or LS columns (Miltenyi Biotec) were calibrated with blocking buffer and loaded with cells. The flowthrough fraction was collected for the assessment of DPP4^−^ cells. The column was then washed three times with blocking buffer and removed from the magnetic board, and DPP4^+^ cells were eluted in 500 µL of blocking buffer. Cells were either pelleted and kept in RLT-DTT buffer for mRNA assessment or cultured in normal growth media for future experiments.

### RNA isolation and RT-qPCR

Whole-tissue samples were lysed for 2 min at 30 Hz/sec in 1 mL of Qiazol (Qiagen) with metal beads in the TissueLyzer II (Qiagen). Samples were kept for 5 min at room temperature, 200 µL of chloroform was added, and samples were incubated for another 3 min at room temperature. Homogenates were centrifuged at 12,000*g* for 15 min at 4°C. Afterward, the clear aqueous phase was transferred to a fresh tube, mixed with the same volume of 70% ethanol, and transferred to RNeasy mini spin columns. For RNA isolation from preadipocytes and differentiated adipocytes, cell pellets were lysed in RLT buffer containing 25 µM dithiothreitol (DTT). Samples were then mixed with the same volume of 70% ethanol and transferred to RNeasy mini spin columns. RNA isolation was performed following the manufacturer's recommendations using a RNeasy mini kit (Qiagen), except for DPP4 sorted cells, where an RNeasy micro kit was used. RNA yield was determined using a NanoDrop 2000 UV-Vis spectrophotometer (Thermo Scientific).

To determine gene expression, cDNA (200–500 ng of total RNA) was synthesized using the high-capacity cDNA reverse transcription kit (Applied Biosystems). Relative mRNA expression was quantified by mixing cDNA, 300 nM forward and reverse primers, and iTaq Universal Green Supermix (Bio-Rad) and running the mixture in a C1000 Touch thermal cycler (Bio-Rad). Samples were run in duplicates or triplicates for each gene and quantified using the Bio-Rad CFX Manager 3.1 program. All expression levels were normalized to TATA-binding protein (*Tbp*). *Cq*-value was set to 50 when there was no gene amplification but *Tbp* expression was detected. Primer sequences used for the analysis were as follows: *Igf2* (Fwd: 5′-AGACATACTGTGCCACCCC-3′ and Rev: 5′-ATTGGAAGAACTTGCCCACG-3′), *Fabp4* (Fwd: 5′-GATGCCTTTGTGGGAACCT-3′ and Rev: 5′-CTGTCGTCTGCGGTGATTT-3′), *Pparg* (Fwd: 5′-CCCTGGCAAAGCATTTGTAT-3′ and Rev: 5′-GAAACTGGCACCCTTGAAAA-3′), *AdipQ* (Fwd: 5′-GATGGCACTCCTGGAGAGAA-3′ and Rev: 5′-TCTCCAGGCTCTCCTTTCCT-3′), *Tbp* (Fwd: 5′-ACCCTTCACCAATGACTCCTATG-3′ and Rev: 5′-TGACTGCAGCAAATCGCTTGG-3′), *Dpp4* (Fwd: 5′-TCAGCTCATCCTCTAGTGCG-3′ and Rev: 5′-AGCCCACACCACATCACATA-3′), *Ki67* (Fwd: 5′-CCTTTGCTGTCCCCGAAGA-3′ and Rev: 5′-GGCTTCTCATCTGTTGCTTCCT-3′), *Igf1r* (Fwd: 5′-ATCGCGATTTCTGCGCCAACA-3′ and Rev: 5′ -TTCTTCTCTTCATCGCCGCAGACT-3′), and InsR (Fwd: 5′-AAATGCAGGAACTCTCGGAAGCCT-3′ and Rev 5′-ACCTTCGAGGATTTGGCAGACCTT-3′).

### Protein isolation and Western blot

Differentiated preadipocytes or cultured primary prewean preadipocytes that were serum-starved for 24 h in the presence of 1 µg/mL IgG or IGF2-neutralizing antibody were washed with ice-cold PBS and lysed using RIPA buffer (50 mM Tris at pH 7.4, 150 mM NaCl, 1 mM EDTA, 1% Triton X-100) containing 0.1% sodium dodecyl sulfate (SDS), 0.01% protease inhibitor, and 0.01% phosphatase inhibitor cocktails II and III (all Sigma). Lysates were incubated for 10 min on ice and then centrifuged at 14,000*g* for 10 min at 4°C. Protein concentration was determined by BCA protein assay kit (Thermo Scientific). Samples were mixed with NuPAGE sample buffer (Life Technologies) containing 2.5% β-mercaptoethanol (Carl ROTH) and heated for 5 min at 95°C. Samples were then loaded on a SDS-PAGE gel with a Fisher BioReagents EZ-Run Prestained Rec protein ladder (Thermo Scientific) and transferred to 0.45 µm PVDF membranes (Merck Millipore). Unspecific binding sites were blocked using 5% bovine serum albumin (BSA) or nonfat dried milk in TBS-T (1× TBS containing 0.1% Tween-20) for 1 h at room temperature, followed by incubation with the following primary antibodies overnight at 4°C: AKT (1:1000; Cell Signaling Technology 4685), phospho-AKT Ser 473 (1:1000; Cell Signaling Technology 9271), ERK (1:1000; Cell Signaling Technology 4695), phospho-ERK (1:1000; Cell Signaling Technology 4377), insulin receptor (InsR; 1:1000; Cell Signaling Technology 3020), IGF1 receptor (IGF1R; 1:1000; Cell Signaling Technology 9750), PPARg (1:1000; Cell Signaling Technology 2443), and B-Actin HRP (only 30 min at room temperature; 1:5000; Santa Cruz Biotechnology sc-47778). The next day, the membrane was washed with TBS-T and incubated with the following secondary antibodies for 1 h at room temperature: antirabbit HRP (1:5000; Cell Signaling Technology 7074) and antimouse HRP (1:5000; Santa Cruz Biotechnology sc-2005). After washing, the membrane was incubated with either ECL (Merck Millipore) or SuperSignal ECL (Thermo Scientific) for 1 min and imaged by the ChemiDoc (Bio-Rad).

#### Immunoprecipitation (IP)

Cultured primary scWAT preadipocytes were serum-starved for 3 h, stimulated with 10 nM IGF2 for 10 min, and lysed in IP lysis buffer (RIPA with 2 mM EDTA, 0.01% protease inhibitor, 0.01% phosphatase inhibitor cocktails II and III). Protein isolation was the same as mentioned above. Two-hundred micrograms of protein lysates was incubated with either anti-InsR or anti-IGF1R antibodies (both 1:100) overnight at 4°C with gentle rotation. The next day, lysate–antibody mixes were incubated with 15 µL of A/G agarose beads for 1–2 h at 4°C and washed three times with IP lysis buffer. The immune complex was eluted by incubating the lysates with 2× NuPAGE sample buffer (Life Technologies) containing 5% β-mercaptoethanol and boiling for 5 min at 95°C. Samples were run on a NuPAGE 4%–12% Bis-Tris gel (Invitrogen) in 1× MOPS buffer at 200 V and transferred to 0.45 µm PVDF membranes (Merck Millipore). The membrane was blocked with 5% BSA and incubated with the following primary antibodies overnight at 4°C: phospho-tyrosine (1:1000; Cell Signaling Technology 8954), InsR (1:1000; Cell Signaling Technology 3020), IGF1R (1:1000; Cell Signaling Technology 9750), and B-Actin HRP (only 30 min at room temperature; 1:5000; Santa Cruz Biotechnology sc-47778). The next day, the membranes were washed with TBS-T and incubated with the following secondary antibodies for 1 h at room temperature: antirabbit HRP (1:5000; Cell Signaling Technology 7074) and antimouse HRP (1:5000; Santa Cruz Biotechnology sc-2005). After the washing steps, the membrane was incubated with SuperSignal ECL (Thermo Scientific) for 1 min and imaged using the ChemiDoc (Bio-Rad).

#### RealTime-Glo MT cell viability assay (proliferation)

The RealTime-Glo MT cell viability assay was performed according to the manufacturer's instructions (Promega G9711). Briefly, cultured DPP4^+^ and DPP4^−^ preadipocytes were seeded in 96 well plates at a density of 1 × 10^4^ cells per well. A mixture of MT cell viability substrate and NanoLuc enzyme was added to the media in the presence or absence of 10 nM IGF2 or 1 µg/mL IGF2-neutralizing antibody. Luminescence was measured daily using a PHERAstar FSX (BMG Labtech) for up to 7 days. Data are expressed as luminescent signals after subtraction of background values determined from the culture medium.

### MTT assay

Isolated scWAT preadipocytes were grown to 80% confluence and seeded on 96 well plates with 1 × 10^4^ cells per well. A plate was prepared for each day of the measurement. Cells were incubated with 0.5 mg/mL final concentration MTT in the well for 2 h at 37°C, except on the seeding day, when cells were incubated for 3 h after seeding with MTT. Death control wells were treated with 0.03% Triton X-100 for 1 min, and then MTT was added to the wells. Formazan was eluted with solubilization solution (10% Triton X-100, 0.03% HCl in 100% isopropanol) with shaking at 700 rpm for 10 min at room temperature. The supernatant was then transferred into a fresh 96 well plate (Greiner, F-bottom), and its absorbance was measured at 570 nm using either a Varioskan LUX multimode plate reader (Thermo Scientific) or a PHERAstar FSX (BMG Labtech). Background absorbance was measured at 640 nm. The preadipocytes of adult mice were supplemented with 10 nM IGF2 starting from the first day of seeding until day 7 and cultured in 1% adult MS (d0–d7).

### Fluorescent in situ hybridization by RNAscope

RNAscope staining (Bio-Techne 323133) of mm-Igf2 (Atto 570; Bio-Techne 437671) was performed on 20 µm cryosections of prewean and adult scWAT according to the manufacturer's instructions. Two micrometer paraffin sections of prewean scWAT with skin were stained for mm-Igf2 and TSA vivid fluorophore 570 (Bio-Techne PN 323272) following the manufacturer's manual for the RNAscope (Bio-Techne 323100), except that the antigen retrieval was for 30 min. RNAscope 3-plex positive (Bio-Techne 320881) and negative (Bio-Techne 320871) probes were used as control stainings. Sections were subsequently blocked with 10% goat serum for 1 h at room temperature and stained for DPP4 (1:1000; Abcam ab187048) in 5% goat serum overnight at 4°C. Slides were washed three times with PBS for 5 min at room temperature and stained with the following secondary antibodies for 1 h at room temperature: donkey antirabbit Alexa 488 (1:400; Invitrogen A21206) and WGA-conjugated Alexa 647 (1:1000; Invitrogen W32466). After the slides were washed three times with PBS for 5 min, sections were incubated with DAPI for 30 sec and mounted with DAKO mounting media. Fluorescent signal was imaged using a SP8 confocal microscope (Leica).

### ELISA

Serum levels of insulin and IGF2 were measured using ultrasensitive mouse insulin ELISA kits (Crystal Chem 90080) and m/r/p/calIGF-II Qkit (R&D Systems MG200), respectively, following the manufacturer's instructions.

### Immunocytochemistry

In vitro differentiated primary adipocytes were fixed with 10% formalin for 10 min at room temperature, permeabilized with 0.1% Triton X-100 on ice, and blocked with 3% BSA containing 0.03% Triton X-100 for 1 h at room temperature. Slides were stained for Alexa 647-conjugated phalloidin (1:40; Life Technologies A22287) and LipidTox Green (1:200; Life Technologies H34475) in blocking buffer for 1 h at room temperature and washed three times with PBS. Sections were mounted using Dako fluorescence mounting medium, and images were acquired using a SP8 confocal microscope (Leica).

### Oil Red O (ORO) staining

In vitro differentiated primary preadipocytes were fixed with 10% formalin at room temperature. Cells were dehydrated with 60% isopropanol for 5 min and dried. Cells were incubated with ORO working solution (60% stock diluted in ddH_2_O) for 10 min at room temperature and washed with ddH_2_O. Cell number was measured after staining cells with DAPI at 1:5000 for 5 min and measuring fluorescence intensity at 460 nm using PHERAstar FSX (BMG Labtech). ORO was diluted in 100% isopropanol, and absorbance was measured at 500 nm using PHERAstar FSX.

### Statistical analysis

Statistical significance for multiple comparisons was determined by ordinary one-way ANOVA or two-way ANOVA with Tukey's multiple comparisons test or unpaired two-tailed *t*-test using GraphPad Prism 10.0.2. All the statistical tests for the scRNA-seq experiments were performed in R, and the bar plots were generated using the ggplot2 package. All the respective *P*-values in violin plots were calculated using the Wilcoxon test and stat_compare_means function from the ggpubr package for respective condition pairs. Data are shown as ±standard error of the mean (SEM). Exact *P*-values are indicated in the figures, and *P* < 0.05 was considered statistically significant.

## Supplemental Material

Supplement 1
