# Supplementary material for: Igf2 regulates early postnatal DPP4+ preadipocyte pool expansion
Source: Genes Dev. 2025 Dec 1;39(23-24):1414–25. doi: 10.1101/gad.352710.125 (PMC12667387; doi:10.1101/gad.352710.125)
Supplement: Supplement 1 [file Supplemental_Figures.pdf]

Supplemental Figures

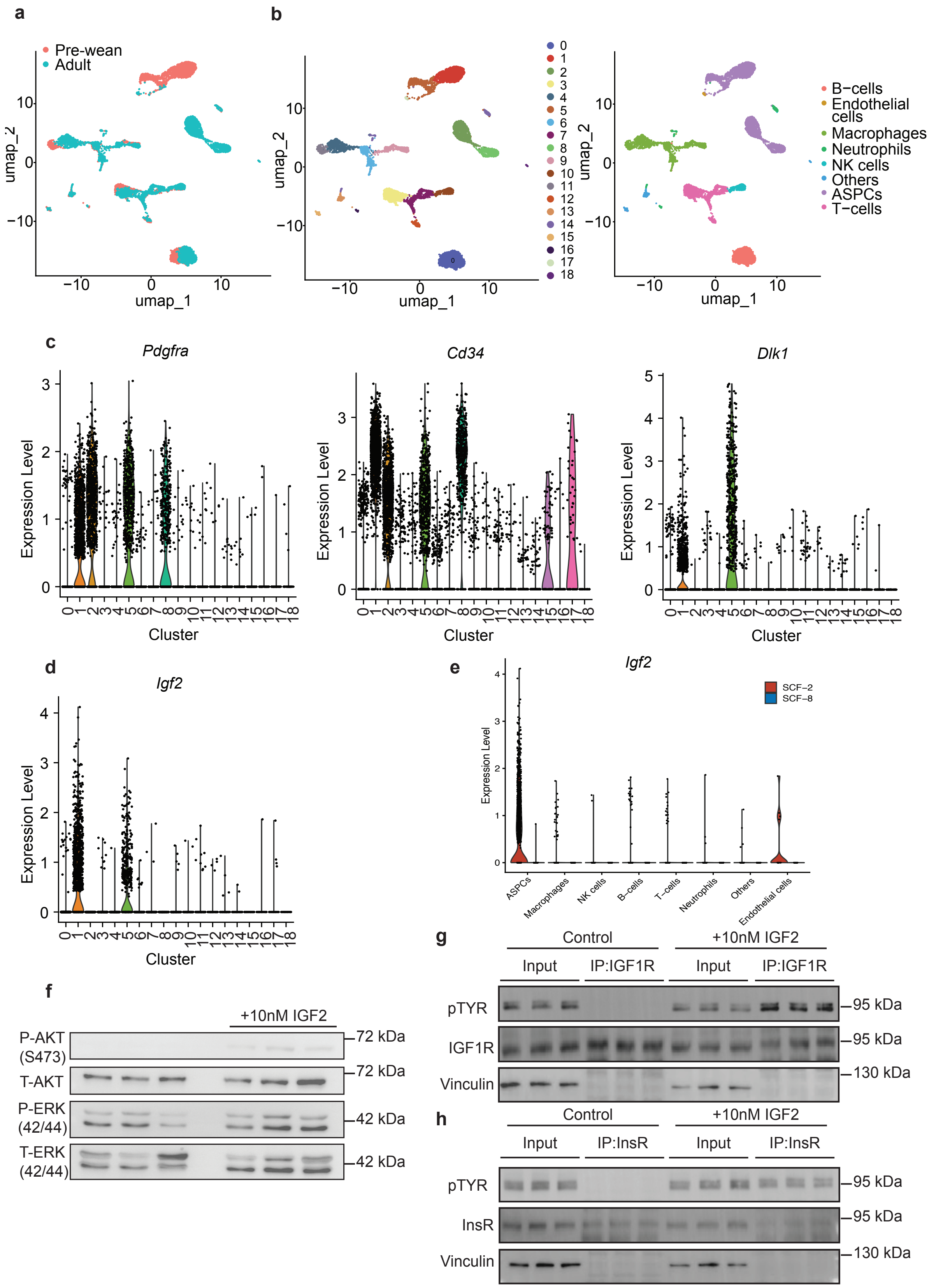

Supplemental Figure S1: IGF2 activates AKT and ERK through IGF1R and InsR signaling pathways. (**a**) Projection of two age groups (pre-wean and adult) on isolated scWAT SVCs shown as umap plot. (**b**) Cell Clustering of (a) based on cell types. Violin plot of (**c**) preadipocyte markers and (**d**) *Igf2*. (**e**) Igf2 expression in different cell types assigned in (b).(**f**) Adult primary scWAT preadipocytes were serum starved 3h and stimulated with 10 nM IGF2 for 10 min. Western blot of phosphorylated (P)/ total (T) AKT and ERK (n=3). Immunoprecipitation (IP) of (**g**) IGF1R (n=3) and (**h**) InsR (n=3). Immunoblotting of p-Tyrosine (TYR) was used to assess the phosphorylation of the receptors after IGF2 stimulation.

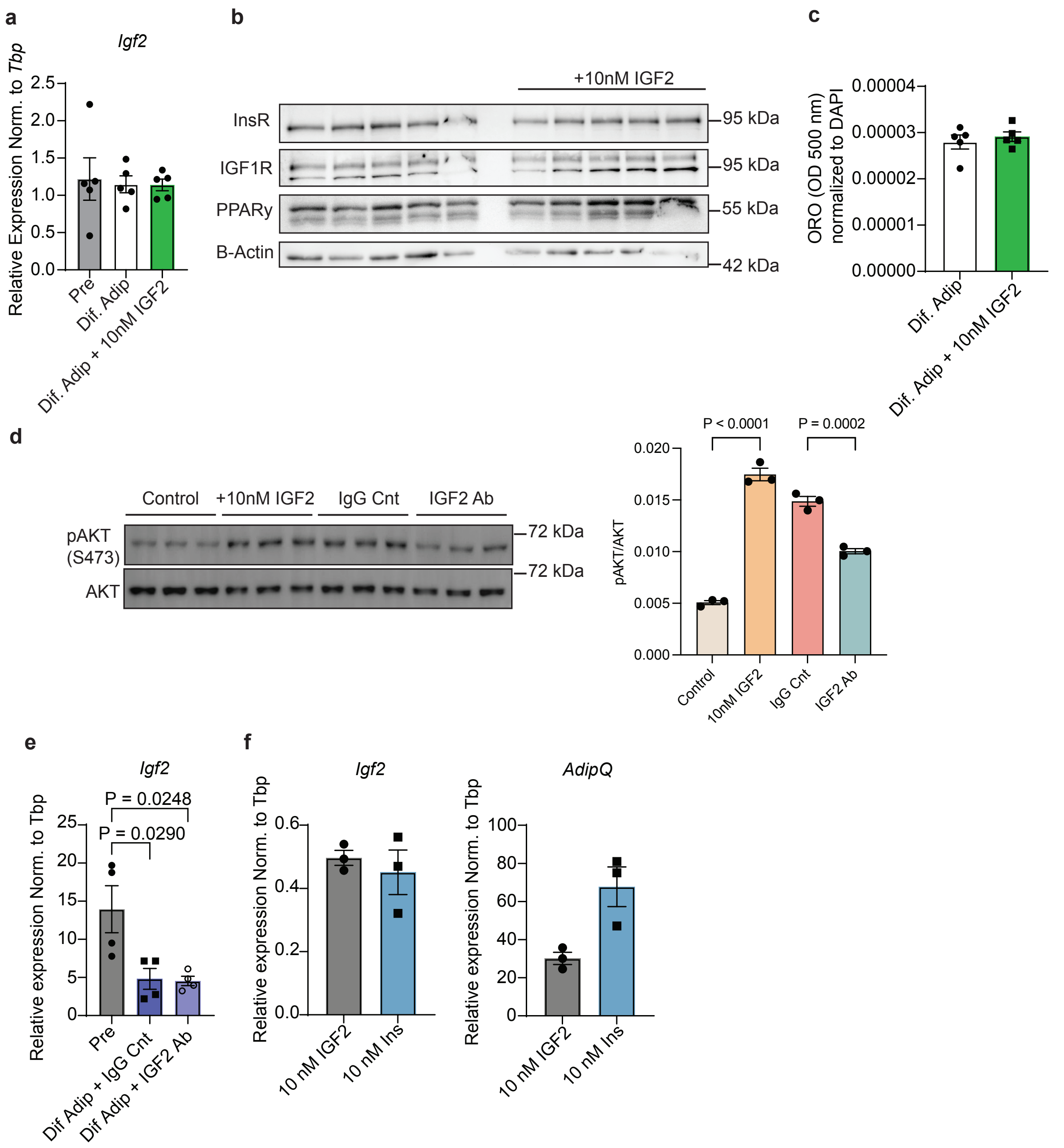

Supplemental Figure S2: Cultured adult scWAT preadipocytes (Pre) were differentiated with (Dif. Adip + 10nM IGF2) or without (Dif. Adip) 10 nM IGF2. (**a**) mRNA expression of *Igf2* (n=5). (**b**) Western blot of InsR, IGF1R and PPARg. B-Actin was used as loading control (n=5). (**c**) Lipid quantification by Oil red O (ORO) absorbance at 500 nm normalized to DAPI of only differentiated primary adult scWAT preadipocytes (n=5). (**d**) (left) Western blot of phosphorylated (pAKT(S473)) and total AKT of protein isolated from cultured adult scWAT ASPCs that were serum starved for 3h followed by stimulation with 10nM IGF2 in the presence or absence of 1µg/ml IgG or IGF2 neutralizing antibody (IGF2 Ab) for 24h (n=3). (right) Quantification of pAKT normalized to total AKT. (**e**) mRNA levels of *Igf2* from differentiated

pre-wean scWAT primary preadipocytes that were treated with 1 μg/ml IgG (Dif. Adip + IgG Cnt) or IGF2 neutralizing antibody (Dif. Adip IGF2 Ab) (n=4). (**f**) 100 nM insulin was substituted with either 10 nM IGF2 or insulin during differentiation. mRNA levels of *Igf2* and *AdipQ* (n=3). Data shown by mean ± SEM.

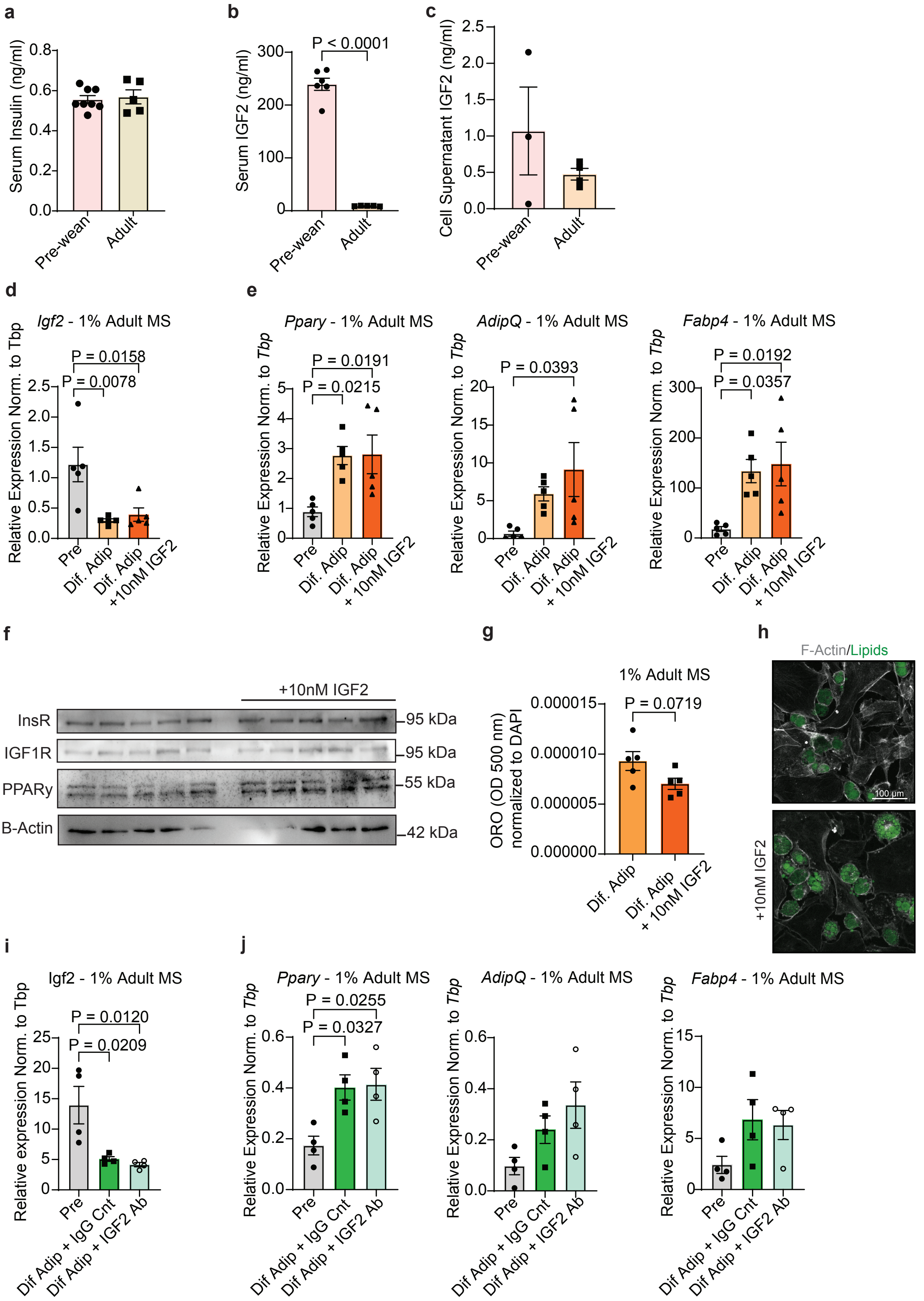

Supplemental Figure S3: IGF2 does not alter differentiation of preadipocytes despite the substitution of FBS with mouse serum. Circulating levels of (**a**) insulin (n=8 pre-wean and n=5 adult) and (**b**) IGF2 (n=6 pre-wean and n=5 adult) in pre-wean and adult mice. (**c**) Secreted

IGF2 levels in cell culture supernatant of pre-wean and adult primary scWAT preadipocytes (n= 3 pre-wean and n=4 adult). Primary adult preadipocytes were supplemented with (Dif. Adip + 10 nM IGF2) or without (Dif. Adip) 10 nM IGF2 during differentiation. mRNA expression levels of (**d**) *Igf2* (n=5) and (**e**) adipogenic markers (n=5). (**f**) Western blot of differentiated adipocytes for InsR, IGF1R, PPARg. B-Actin was used as loading control (n=5). (**g**) Lipid quantification by Oil Red O (ORO) (n=5). (**h**) Immunocytochemistry of F-Actin (grey) and Lipids (green) (n=4). Primary pre-wean preadipocytes were treated with 1 µg/ml IgG (Dif. Adip + IgG Cnt) or IGF2 neutralizing antibody (Dif. Adip IGF2 Ab) during differentiation. MRNA expression levels of (**i**) *Igf2* (n=4) and (**j**) adipogenic markers (n=4). Data shown by mean ± SEM.

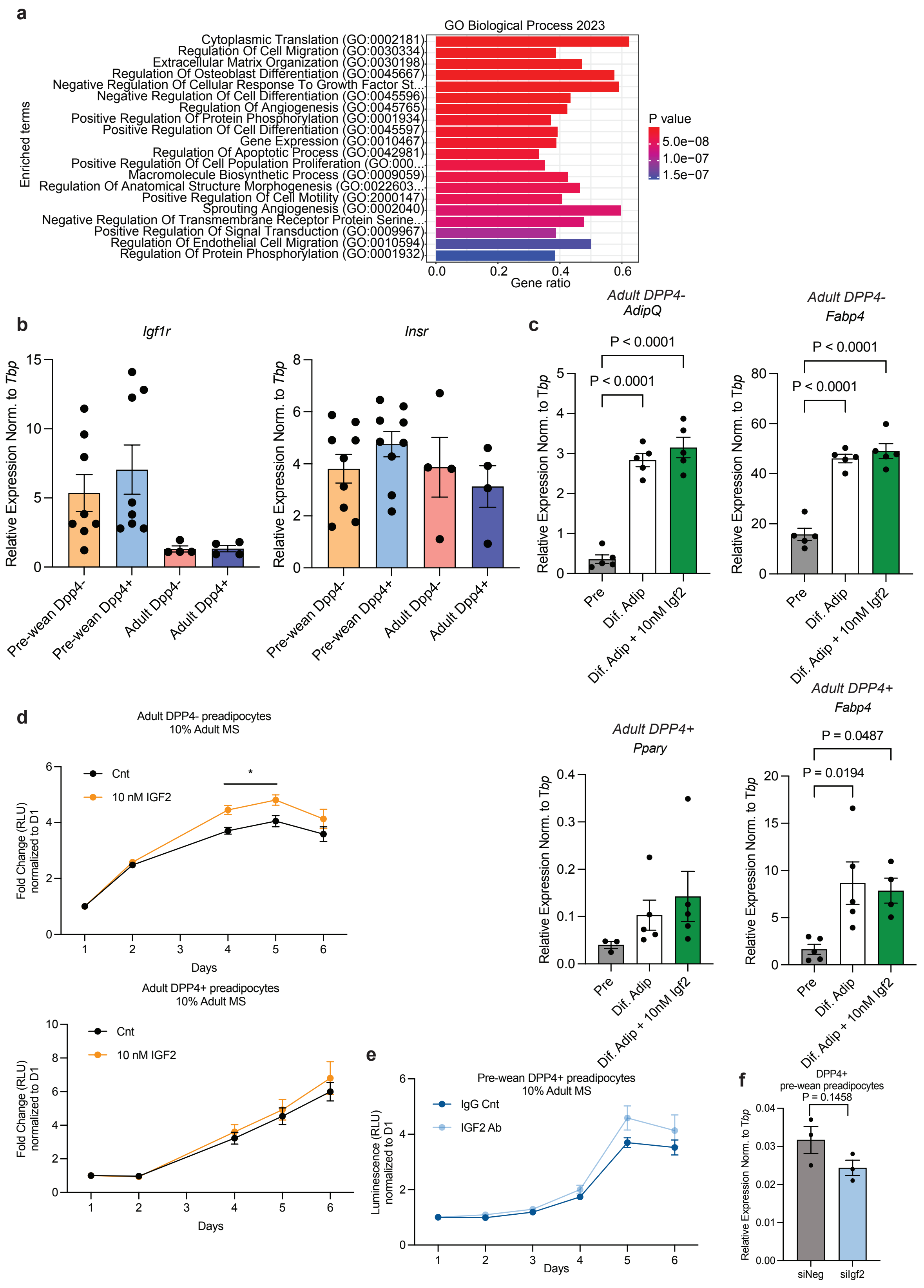

Supplemental Figure S4: *Igf2* enriched cells express genes regulating ECM organization and cell proliferation. (**a**) Gene enrichment analysis of *Igf2* enriched vs low cell clusters from Fig. 3b for Gene Ontology (GO) Biological Process. (**b**) mRNA expression of *Igf1r and Insr*

in MACS sorted DPP4+ and DPP4- pre-wean and adult SVCs. (**c**) mRNA levels of adipogenic markers in MACS sorted DPP4- and DPP4+ adult SVCs in the supplement of 10nM IGF2 (n=5). Pre: undifferentiated cells, Dif. Adip: Differentiated Adipocytes. Data shown by mean ± SEM. (**d**) RealTime-Glo MT cell assay of MACS sorted DPP4+ and DPP4- adult preadipocytes cultured with 10% adult mice mouse serum and supplemented with or without 10 nM IGF2 for 6 days (n=5). (**e**) RealTime-Glo MT cell viability assay of MACS sorted DPP4+ pre-wean preadipocytes cultured with 10% adult mice mouse serum and treated with 1 µg/ml IgG (IgG Cnt) or IGF2 neutralizing antibody (IGF2 Ab) (n=4).
